# Ontario Veterinary College

**Published:** 1891-07

**Authors:** 


*Ontario Veterinary College.*—The following graduates of the Ontario Veterinary College, have been appointed inspectors of the U. S. Department of Agriculture, Bureau of Animal Industry: Dr. M. Hawkens, Detroit, Michigan; Dr. T. J. Claris, Buffalo, New York; Dr. O. L. Boor, Muncie, Ind.

Dr. T. D. Hinebauch has been appointed Professor of Veterinary Science, Dakota Agriculture College.

